# The frequency of lichenoid features in mucous membrane pemphigoid: A retrospective review of the histopathology

**DOI:** 10.1016/j.jdin.2024.07.007

**Published:** 2024-08-02

**Authors:** Sarah G. McAlpine, Paul B. Googe, Donna A. Culton

**Affiliations:** a University of North Carolina, School of Medicine, Chapel Hill, North Carolina; b Department of Dermatology, University of North Carolina, Chapel Hill, North Carolina

**Keywords:** dermatopathology, direct immunofluorescence, general dermatology, mucous membrane pemphigoid, oral lichen planus, oral mucosa

*To the Editor:* Accurate diagnosis of inflammatory oral mucositis can be challenging given the similar clinical features of underlying conditions including oral lichen planus (OLP), mucous membrane pemphigoid (MMP), and pemphigus vulgaris.^1^ Appropriate therapeutic management depends on distinguishing between these conditions. Biopsy for routine microscopic evaluation with hematoxylin and eosin (H&E) staining often supports diagnosis as each condition has classic histologic features. However, in practice, histologic findings are often nonspecific or overlap, making differentiation difficult, particularly between OLP and MMP.^2^ While direct immunofluorescence (DIF) is recommended in these cases, it is not always performed if histology suggests a particular condition. One European study reported the frequency of lichenoid features in MMP.^3^ Our study aimed to describe the frequency of lichenoid findings in patients with MMP in a United States cohort.

Ninety-five patients with MMP showing linear IgG and C3 basement membrane zone (BMZ) staining on DIF from February 2020 to January 2024 were identified at the UNC immunofluorescence laboratory.

Patient records were reviewed for pathology reports of biopsies submitted for H&E. Thirteen patients without H&Es were excluded. Of 82 patients with MMP, 22.0% had lichenoid features, 48.8% showed subepithelial blistering, vesiculoulcerative, or vesiculobullous findings, 11.0% had ulcerative mucositis, 9.8% had chronic nonspecific inflammation, and 8.5% had other diagnoses (Table I). Oral pathology read 81 cases. One case was read by dermatopathology. Biopsy and disease activity sites were not widely available from the requisition forms, limiting our ability to investigate possible clinical differences in these MMP subsets. OLP was suspected clinically in 11 of the 18 “lichenoid features” cases. A dermatopathologist’s (P.B.G.) blinded review of these 18 H&E slides revealed that 77.8% had a band of lymphocytic inflammation, 33.3% had basal layer vacuolization, 22.2% had focal BMZ thickening, and 61.1% showed subepithelial split (Table II).

**Table I:** Histopathological descriptions of MMP biopsy specimen for H&E

	*N* (%)
Lichenoid features	18 (22.0)
Subepithelial blister, vesiculoulcerative, or vesiculobullous findings	40 (48.8)
Ulcerative mucositis	9 (11.0)
Chronic nonspecific inflammation	8 (9.8)
Other	7 (8.5)

Summary of the histopathological descriptions reported in 82 oral specimens stained by H&E. The diagnoses were grouped into 5 categories: lichenoid features, subepithelial blister, vesiculoulcerative and vesiculobullous, ulcerative mucositis, chronic nonspecific inflammation, and others. The number of cases in each group and the percentage these cases represent are listed.

*H&E*, Hematoxylin and eosin; *MMP*, mucous membrane pemphigoid.

**Table II:** Review of the histopathologic findings of the 18 “lichenoid features” biopsy specimens

	*N* (%)
Band of lymphocytic inflammation	14 (77.8)
Basal layer vacuolization	6 (33.3)
Basement membrane zone thickening	4 (22.2)
Subepithelial split	11 (61.1)

Summary of specific histopathological findings observed by a blinded dermatopathologist in the 18 H&E-stained biopsy specimens reported to have “lichenoid features” in Table I.

*H&E*, Hematoxylin and eosin.

Lichenoid features typically refer to a band-like infiltrate of lymphocytes along the BMZ. When noted in oral inflammatory conditions, lichenoid features may be suggestive of OLP, but alone are not diagnostic and can be seen in numerous oral conditions such as graft versus host disease, chronic ulcerative stomatitis, lichen planus pemphigoides (LPP), intraoral discoid lupus, and dysplasia.^2^ Though OLP is far more prevalent, our data suggests that MMP should be considered in the differential diagnosis of oral conditions with lichenoid findings.^3^^,^^4^ LPP has been described as an overlap condition with clinical and histologic features of both lichen planus and bullous pemphigoid. While oral cases of LPP are reported, the diagnostic criteria are not well described. It is unclear if MMP with lichenoid features represents LPP.^5^

Histopathological findings can guide the diagnosis of oral inflammatory conditions; however, they may be misleading or overinterpreted, particularly those with lichenoid features. This study reports a relatively high frequency of lichenoid features in MMP, highlighting the importance of DIF to distinguish between OLP and MMP in patients with inflammatory mucositis, even when histopathology suggests OLP. Future studies comparing clinical features of lichenoid MMP to OLP would further clarify this MMP histologic subtype.

## Conflicts of interest

None disclosed.
